# Iterative Design and Evaluation of a Tangible Robot-Assisted Handwriting Activity for Special Education

**DOI:** 10.3389/frobt.2020.00029

**Published:** 2020-03-06

**Authors:** Arzu Guneysu Ozgur, Ayberk Özgür, Thibault Asselborn, Wafa Johal, Elmira Yadollahi, Barbara Bruno, Melissa Skweres, Pierre Dillenbourg

**Affiliations:** ^1^Computer-Human Interaction in Learning and Instruction Laboratory (CHILI), EPFL, Lausanne, Switzerland; ^2^University of New South Wales, Sydney, NSW, Australia; ^3^MOBOTS Group of the Biorobotics Laboratory (BIOROB), EPFL, Lausanne, Switzerland; ^4^Center for Learning Sciences (LEARN), EPFL, Lausanne, Switzerland

**Keywords:** handwriting, occupational therapy, tangible robots, iterative design, robots for education, haptic devices, interactive learning, special education

## Abstract

In this article we investigate the role of interactive haptic-enabled tangible robots in supporting the learning of cursive letter writing for children with attention and visuomotor coordination issues. We focus on the two principal aspects of handwriting that are linked to these issues: Visual perception and visuomotor coordination. These aspects, respectively, enhance two features of letter representation in the learner's mind in particular, namely the shape (*grapheme*) and the dynamics (*ductus*) of the letter, which constitute the central learning goals in our activity. Building upon an initial design tested with 17 healthy children in a preliminary school, we iteratively ported the activity to an occupational therapy context in 2 different therapy centers, in the context of 3 different summer school camps involving a total of 12 children having writing difficulties. The various iterations allowed us to uncover insights about the design of robot-enhanced writing activities for special education, specifically highlighting the importance of ease of modification of the duration of an activity as well as of adaptable frequency, content, flow and game-play and of providing a range of evaluation test alternatives. Results show that the use of robot-assisted handwriting activities could have a positive impact on the learning of the representation of letters in the context of occupational therapy (*V* = 1, 449, *p* < 0.001, *r* = 0.42). Results also highlight how the design changes made across the iterations affected the outcomes of the handwriting sessions, such as the evaluation of the performances, monitoring of the performances, and the connectedness of the handwriting.

## 1. Introduction

Handwriting is a complex perceptual-motor skill consisting of visuomotor integration, motor planning, visual-spatial abilities, visual perception, as well as responsiveness to tactile and kinesthetic stimuli (Maeland, 1992; Amundson and Weil, 1996; Feder and Majnemer, 2007). It is a fundamental ability which has a great impact on a wide range of tasks such as communicating and recording our knowledge, emotions, ideas and opinions. Unsurprisingly, it has been shown that handwriting is a critical skill to be acquired for the academic and behavioral development of students (Berninger et al., 1997; Feder and Majnemer, 2007; Christensen, 2009). Hence, there is an ongoing research effort dedicated to empowering students with effective writing skills and highlighting the challenges students face to master handwriting.

In recent years, several studies have been conducted exploring the processes engaged in handwriting and the learning effects of different technologies on the handwriting process. Feder and Majnemer (2007) suggested that handwriting difficulties do not resolve without intervention. Considering that up to 25% of the school-aged population is affected by handwriting difficulties (Smits-Engelsman et al., 2001; Charles et al., 2003), there is a need to develop technologies that support intervention methods for typically developing and high-risk populations. One example where technology can be useful in this domain is the usage of digital tablets to detect handwriting difficulties. They made possible the evaluation not only of the final product of handwriting (the static image), but also its dynamics (Asselborn, T. et al., 2018; Zolna et al., 2019). For example, Pagliarini et al. (2017) used digital tablets to collect data on handwriting ability before handwriting is performed automatically. Thanks to quantitative methods, they could find patterns indicating potential future writing impairments at a very early age. Mekyska et al. (2016) used a supervised learning model to detect dysgraphia. The authors included 54 third-grade Israeli children in the study and used a 10-item Handwriting Proficiency Screening Questionnaire (HPSQ) (Rosenblum, 2008) to identify poor writing.

Rosenblum et al. (2019) in their study of handwriting investigated how certain low-level and high-level processes differ between children with ASD and typically developing children. Their findings have clinical implications which can inspire the development of technologies to help children with executive function deficiencies. These results indicate that the accurate assessment performed by therapists to identify the deficits and to determine the appropriate handwriting intervention customized to the individual have considerable importance.

In a related study, Asselborn, T. et al. (2018) focused on the detection of severe handwriting difficulties such as dysgraphia, using a digital approach that identifies and characterizes handwriting difficulties (Asselborn, T. et al., 2018; Zolna et al., 2019). Their approach was inspired by the original standardized test devised by therapists to detect handwriting difficulties. Their tablet-based test can have direct implications on developing educational technologies for children, either typically developing or with handwriting difficulties. Several other tablet-based applications can be found in the literature that remediate handwriting difficulties; the main advantages of these tablet-based applications is that they allow the display of additional visual information to provide immediate adaptive feedback and instructions to the learner, while capturing the handwriting data accurately to be processed in real-time or afterwards (Yamasaki et al., 1990; Lee and Lim, 2013).

Furthermore, a growing number of studies aim at helping children with developmental disorders by incorporating robots to help handwriting (Chandra et al., 2019; Kim et al., 2019). For instance, the Cowriter project (Hood et al., 2015; Chandra et al., 2019) exploits the social capabilities of a humanoid robot to teach handwriting in an original way. Based on the learning-by-teaching approach, the child becomes the teacher of a robot “requiring help” to improve its handwriting and this role reversal results in several powerful effects including motivation gain and de-dramatization of the child's problems.

From a learning goals perspective, in order to have a complete letter representation, a child should acquire the visual perception of the letter, called the *grapheme*, but also the visuomotor coordination associated with it, i.e., the dynamics of the movement, called *ductus* (Bara and Gentaz, 2011). To enhance the visual perception as well as the visuomotor coordination, it is shown that using more sensory information ranging from audio and visual to kinesthetic feedback is important (Hayes, 1982; Bluteau et al., 2008; Bara and Gentaz, 2011; Danna and Velay, 2015). Because of this reason, teachers commonly use techniques allowing children to experience various sensory information when learning how to write. These techniques include drawing letters in sand or semolina, touching and sensing the shape of letters carved in a piece of wood, verbally describing the letters or building the letter with play-dough (Berninger et al., 1997; Arslan, 2012).

Indeed, kinesthetic real-time feedback is shown to be paramount sensory information needed during the process of handwriting (Laszlo and Bairstow, 1984; Laszlo and Broderick, 1991). To fill this gap in robot-assisted and digital technologies, several recent studies are using haptically active training programs in order to teach handwriting. Bara and Gentaz (2011) compared a visual-haptic to a visual only program to teach five different letters to a group of 21 first-grade children. The authors showed that the combination of visual with haptic information is more efficient than visual only information since it improves both perceptual and visuo-motor skills.

Palluel-Germain et al. (2007) showed the use of visual-haptic feedback to teach handwriting to kindergarten children where they present a device, “*Telemaque*,” that incorporates a programmable force-feedback pen that can be guided along a letter model (which is “*not only static (the shape) but also dynamics (rules of motor production)*”) in order to enhance the visuomotor perception of the letters targeted. In their study, the authors focused on six cursive letters (“a,” “b,” “f,” “i,” “l,” “s”) and showed significant improvement of the handwriting's legibility for all trained letters after the visual-haptic training with respect to the control group.

Garcia-Hernandez and Parra-Vega (2009) proposed a haptic tele-operated training method aiming to improve motor skill acquisition. A master helps an apprentice by showing the desired path (a letter) using a robot end-effector, whose motion is sensed by the learner via the haptic device. The authors showed better and faster learning of motor control compared to the condition using visual information only.

Even though these devices have brought very promising results, a strong limitation to their widespread use comes from their very high cost, that makes them unaffordable for most schools. In addition and to the best of our knowledge, there is currently no haptic system providing collaborative handwriting activities in classrooms, which typically requires one set of equipment per learner. For this reason, one of the goals of this article is to present a system for teaching handwriting that relies on low-cost equipment, while also allowing haptic feedback in single- and multi-participant collaborative learning activities.

Collaborative learning appears in situations where two or more people attempt to learn something together (Dillenbourg, 1999). Even if no general assumption can be made concerning the benefits of collaborative learning (because it is strongly dependent on the designed activity), Kreijns et al. (2003) summarize the positive effects that sometimes arise with collaborative learning as a deeper level of learning, critical thinking, shared understanding, and long term retention of the learned material. Moreover, according to the therapists' feedback in the occupational therapy centers, children may benefit from group therapy sessions by modeling their peers, learning how to cooperate, acknowledging each other's strengths. Lastly, group occupational therapy or group physical therapy may provide beneficial social interaction to children: they can not only communicate their ideas with each other, but also improve their self-esteem by achieving skills and tasks in front of their peers. For these reasons, activities and tasks that are planned for the group session should be fun, flexible, exciting and novel as well as in line with the children's goals, preferences and attitudes to minimize the number of children who refuse to participate or exhibit non-compliant behavior^1^.

**Figure 1:**
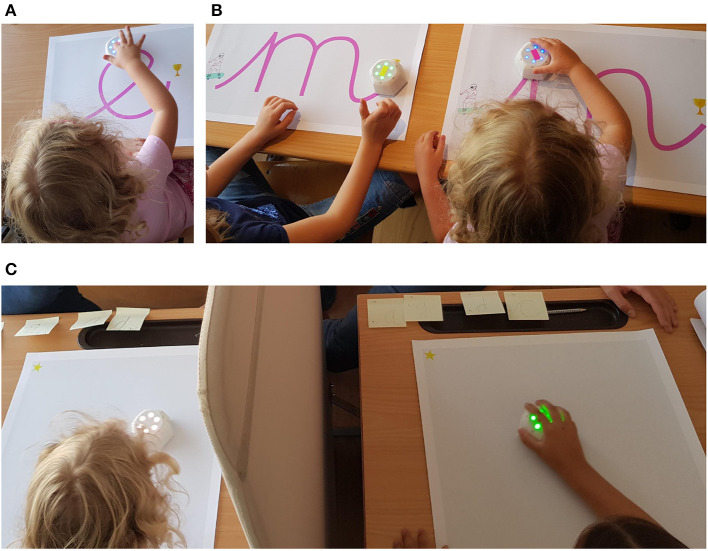
Different sub-activities of tangible robot assisted handwriting activity in a therapy session. **(A)** Feel the robot, **(B)** drive the robot, **(C)** guess the letter game without grapheme.

Our research effort, described in the current and the previous studies (Asselborn, T.* et al., 2018), aims to enhance these sensory information by using the tangible, haptic-enabled, low cost, small-sized Cellulo robots (Özgür et al., 2017). While these robots move on a sheet of paper displaying the letter's visual representation (see Figure 1A), the learner can observe the ductus of the letter (the trajectory followed by the robot between the starting and ending points of the letter), as well as the grapheme of the letter (printed directly on the sheet of paper on which the robot moves). Moreover, the haptic and visual capabilities of the robots allow for increasing the sensory information provided to the learner during the activity. In this article, we hypothesize that training with the robot can effectively convey the procedural knowledge of the grapheme and the ductus of the letter in our context of interest. At the same time, using multiple robots and their synchronized behaviors, we aim to show that it is possible to design collaborative learning activities in the aforementioned fun, flexible and inclusive manner.

The primary aim of this article is to support handwriting learning, with a specific focus on special education, by designing tangible robot-mediated, interactive, collaborative activities. In previous work, we performed a content analysis to target specific skills involved in the handwriting processes and based on that designed an activity flow composed of 4 sub-activities, which was tested and validated in a public school. In this article, we refine and adapt the activity to a therapy context over a number of experiments in different therapy centers, with the close collaboration of therapists and children in need of occupational therapy.

During this iterative design process, we identify the key design aspects to be taken into consideration when addressing occupational therapy scenarios and evaluate the effect of the tested variants on learning using qualitative and quantitative methods. We discuss several key take-home lessons and conclude with shortcomings and future work.

## 2. Materials and Methods

### 2.1. Cellulo Robotic Platform

Cellulo robots are low-cost, small-sized tangible mobile robots that can operate on printed sheets of paper covered with a dot pattern that enables fast (>90 Hz) and accurate localization (sub-mm) of each robot without any calibration (Hostettler et al., 2016; Özgür et al., 2017). This design allows for the recording of rich interaction-related information during the activity, such as user's motion trajectory, accuracy of the motion, etc. The robot's holonomic motion system provides autonomous motion capability, as well as robustness against human manipulation (Özgür et al., 2016). The overall design of the robot allows easy set-up and use in classroom and therapy environments thanks to the plug-and-play nature of its ecosystem. The proposed writing activity is composed of Cellulo robots and several shapes printed on paper sheets, displaying letters and cues related to the letter's ductus (see Figures 1A,B). The haptic, audio, visual and synchronization capabilities of the Cellulo robots allow us to provide real-time multi-sensory feedback during the handwriting task at the individual learner level as well as at the group level during collaborative handwriting activities. Lastly, each robot can be programmed to have a passive, active or semi-active role, which helps us design a pool of different activities where the role of the children can switch in between active and passive.

### 2.2. Iterative Design Methodology

The design of learning activities for children requiring special education within an occupational therapy session brings about many challenges and unknowns, such as orchestration, use of space and choice of grouping of children with vastly different learning objectives and activities. Furthermore, the design of activities for special education involves the crucial participation of a wide spectrum of stakeholders, including teachers, therapists and the children themselves. Given these factors, it becomes impractical to imagine a classical study scenario where a working design can be made and tested successfully to show that it yields positive learning outcomes: As we show below, there are typically many failures, lessons that must be learned and interactions that must be made with the stakeholders in order to improve the existing design and bring it to an acceptable level of operability and adaptability.

For these reasons, we opted to follow an iterative design methodology where we tested and improved the design repeatedly at different stages of maturity and practicality. At each iteration, we make/refine the design and attempt to verify it rigorously with a study in order to reveal flaws and gather observations that may aid in improving it further. First, we start our design by aiming to meet the learning objectives with healthy children in a typical school environment. This is labeled as Iteration 1, and aims to yield a base-level usable activity that we can iterate over; this iteration is previously published in Asselborn, T.* et al. (2018). This also allowed us to eliminate the usability flaws before launching the activity in a therapy environment. In section 3, each iterative step of our iterative design methodology is explained in detail to reflect the design changes, feedback and observations affecting the next steps.

### 2.3. Participants

During the iterative design process, one public school and two therapy centers were involved in our activity design and evaluation. Initial design and evaluation were done in the public school with the contributions of teachers and the participation of 17 healthy children with a mean age of 5.5. During the integration of the system into the therapy setting, therapists of each center gave feedback on the application before the testing stage. In the first center, we conducted one training session with 5 boys. In the second therapy center, we first conducted 3 training sessions with 3 girls and then 2 training sessions with 4 boys. The children attending the sessions had a variety of problems such as difficulty in concentration, fine motor dexterity issues, poor attention, etc. The detailed information about the symptoms and problems of each child indicated by the corresponding therapists of each group can be seen in Table 1. The clinical and neuropsychological assessment data belonging to the participant children are provided directly by the therapists. These assessments include proper clinical diagnoses (reported as ASD and ADHD), but also other potential problems related to handwriting observed by the therapists. These problems may simply be outside of clinical diagnosis scope (reported as e.g., “does not like to write”) or may potentially eventually lead to the discovery of clearly diagnosable disorders in the child (reported as e.g., “motor coordination/activity problems”). In the latter case, the clinical diagnoses were not yet attempted on the children by their legal guardians. We opted to report all of these cases as they were highly beneficial in being the primary guiding factor in both the design phase and the application phase, i.e., when the actual interaction with the affected child took place.

**Table 1:** Child participants to occupational therapy sessions.

**Group**	**Child id**	**Age**	**Symptoms or problems indicated by the corresponding therapists**
Group 1	F	7	ASD, losing motivation quickly, problems in visual construction, does not like to write
	A	7	ADHD, attention problem, sensitivity to auditive stimulation
	X	6	ADHD, attention problem, does not like to write
	B	7	Visuomotor coordination problems, poor fine motor dexterity, problems in line following
	V	7	Visuomotor coordination problems, poor fine motor dexterity, problems in line following
Group 2	J	7	Handwriting problems, poor fine motor skills, poor precision, functional problems, high intelligence assessment, moves a lot and is disturbed quickly, poor concentration
	C	8	Problems in fine motor skills, poor attention, not totally concentrated, focused or engaged while handwriting
	I	5.5	Poor gross and fine motor skills, robot activity is first experience with cursive letters
Group 3	O	7	Problems in handwriting skill and fine motor activity, difficulty in visual perception, line following and drawing
	S	7	Handwriting problems
	K	8	Hyperactive, sensory problems
	M	7	High potential, fine motor skill difficulties, handwriting problems, hyperactive

Testing of our system was part of the three different summer school camps for fine motor and handwriting skills. These camps were aimed at helping with different aspects of handwriting and included varying activities to assist: (1) Core body strength and shoulder stability, (2) Body posture and hand positioning, (3) Manual dexterity and pencil grasp, (4) Fluidity of writing movements, (5) Handwriting legibility, (6) Typing, (7) Sensory awareness, (8) Graphomotor skills, (9) Concentration and attention, (10) Social skills. In the second therapy center, therapists were also providing support for the development of gross motor skills with outdoor activities.

This study was carried out in accordance with the recommendations of the Human Research Ethics Committee (HREC) at EPFL. The protocol was approved by the HREC (No. HREC 008-2018/16.02.2018). All subjects' parent or legal guardian gave written informed consent in accordance with the Declaration of Helsinki. All child participants gave a recorded assent and were informed of their right to stop the experiment at any time.

### 2.4. Data Analysis

In order to explore the added value of our robot-assisted writing activities to the handwriting learning process, we want to assess the visual perception (representation of the letter's grapheme) and the visuomotor coordination (representation of the letter's ductus) aspects of the learners in detail. In other words, we want to assess the quality of the letter representation in the child's mind in terms of ductus and grapheme.

Children participated in each activity session in the following way: First, they did a pre-test with a pressure-sensitive pen & tablet (Wacom Cintiq Pro in the public school, Lenovo ThinkPad X1 Yoga in the therapy centers) in order to measure their handwriting proficiency before the activity. Then, they participated in the tangible robot-enabled activity, namely the main writing session with the Cellulo robots. Finally, they did a post-test in a similar way to the pre-test to measure their progress after our activity.

Initially, we asked experts to grade each letter from every child in terms of the ductus quality between 0 (for totally wrong ductus) and 3 (perfect ductus with proper start and end points and directions) but the inter-rater agreement of the experts was found to be too low. One of the contributing factors was the high variance between the hand writing performance (ductus, grapheme and cursiveness quality) of children in therapy centers. Another was that during the initial phases of the experiment, tests were mis-perceived by some children who started to fill the letter graphemes as if it was a line following (in the form of painting) activity rather than a writing activity. Ranking between 0 and 3 was also not reflecting the improvement in writing performance of children who were previously unable to write at all: There were instances where the pre-test performance was not gradeable (no sensible letter was written) and the post-test performance was very low but comparably closer to actual writing. Some learning clearly took place, but both performances received 0 rank.

In order to reliably quantify the letter writing performance by focusing on grapheme and ductus quality, we switched to the Dynamic Time Warping (DTW) technique from Salvador and Chan (2004) (available as a python package under the name of fast-dtw) which allows measuring the distance between two temporal sequences regardless of the speed. Using this technique, we measured the distance between the written letters [taken as an actual time series (*x, y, t*)] and the ideal letter represented on the activity sheets [taken as an ideal imaginary time series (*x, y, t*)], which is taken as a factor contributing to performance. This distance is used as an error score for writing performances. For a given letter, a lower error score indicates a closer ductus and grapheme to the expected letter. Furthermore, we calculated the connectedness of the letters (defined as the total number of strokes per letter) as another factor contributing to performance, in order to take into account the possible mis-perception effect mentioned above.

## 3. Iterative Design of the Robot-Assisted Writing Activity

**Figure 2:**
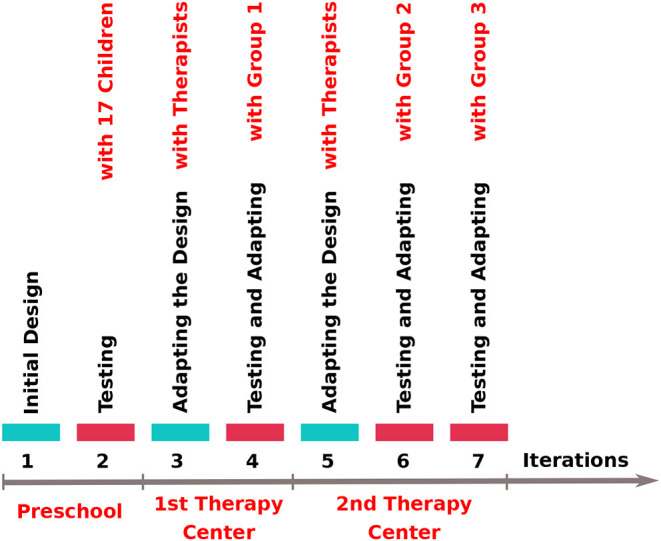
The timeline of the iterative design and testing steps. Group 1, Group 2, and Group 3 are child participants in occupational therapy sessions in Table 1.

This section explains in detail each step through the iterative design process, starting from the pedagogical design, followed by the various steps of testing in the school and therapy centers, and the adaptation of the system to the new learning environment. The overall flow of the iterations and corresponding group information can be seen in Figure 2.

### 3.1. Initial Design of the Letter Writing Activity: Iteration 1

#### 3.1.1. Pedagogical Design

In the initial design, previously published in Asselborn, T.* et al. (2018), our focus was on enhancing the knowledge of the grapheme and the ductus of the letter which are correlated with the visual perception and the visuomotor coordination. The content analysis done to determine the specific skills involved has led us to define the following sub-goals:

- *Remembering the Grapheme*: Memorizing the letter's physical representation (Free Recall and Recognition).
- *Remembering the Ductus*: Memorizing the letter's drawing pattern (Imitation).
- *Remembering the Phoneme to Ductus-Grapheme Link*: Memorizing the link between the letter's pronunciation (phoneme) and the corresponding grapheme and ductus.

It is shown that using more sensory information ranging from audio, visual to kinesthetic feedback enhances visual perception as well as the visuomotor coordination (Hayes, 1982; Bluteau et al., 2008; Bara and Gentaz, 2011; Danna and Velay, 2015). Precisely because of this, teachers use techniques allowing children to experience various sensory information during letter learning such as using sand filled boxes for drawing letters in; touching and sensing the grapheme of letters craved in a piece of wood or plastic surface^2^; or building the letter with play-dough or with similar materials that can be shaped by hands (Berninger et al., 1997; Arslan, 2012). There also exist sensory play games used in therapy centers such as *draw on your back* game. Each child takes turns with the teacher or therapist in drawing with their finger on the other's back. The main goal is to try to guess what the other person is drawing or writing. The level of difficulty is easily adjusted by modifying what is drawn - starting with shapes for young children, progressing through letters of their name, numbers, and so on^3^. The design of our robot-mediated activity is inspired from these traditional methods that are already used in classrooms, as well as from discussions with school teachers and therapists on how we can position Cellulo in handwriting activities.

#### 3.1.2. Activity Design

**Figure 3:**
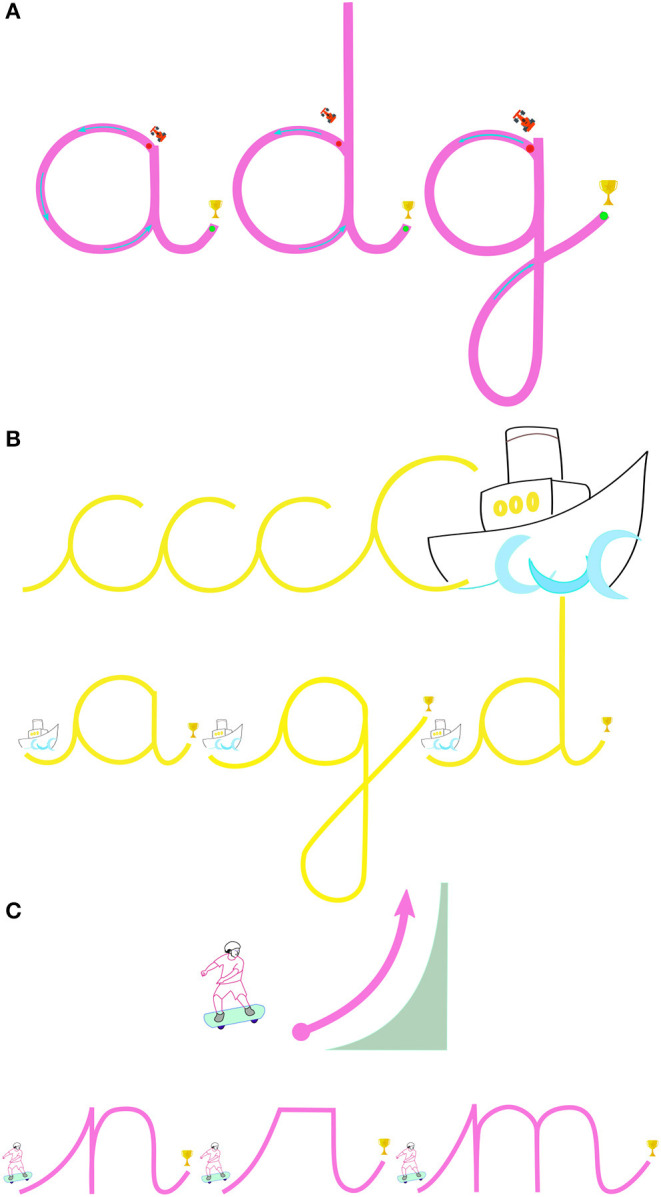
**(A)** Includes round letter maps with the racing theme, letter “a” used in the public school (Iterations 1 and 2) and all three letters are used in first therapy center (Iterations 3 and 4). **(B,C)** Are cursive letters and their initial strokes for practice used in the second therapy center (Iterations 5, 6, and 7). **(B)** Includes examples of wave letters, **(C)** includes examples of skateboarding letters. Adapted from the ABC Boum + teaching approach of graphomotricity.

**Figure 4:**
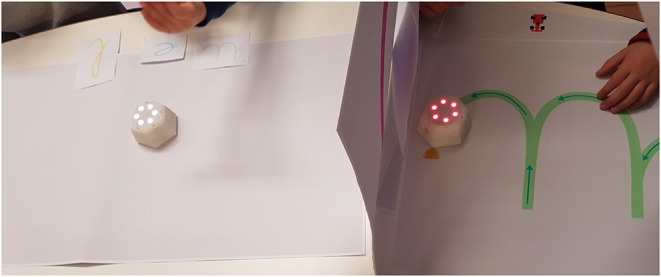
Guess the Letter Game with Grapheme: The children on the left side of the barrier are the guessers and the child on the right side is the writer who just finished writing ‘u’ with the map having grapheme of the letter and waiting for the guessers to guess the written letter.

We decided to use three features of the robot, namely haptic information, autonomous motion and synchronized behavior of multiple robots, to increase the multi-sensory feedback via touch, motion and sight. Haptic features allow each child to receive individual real-time feedback, autonomous motion makes the robot reproduce the ductus while synchronization allows collaborative game design. With this in mind, we designed the following sequence of sub-activities:

- Sub-Activity 1: Link between grapheme and ductus - *Watch the Robot* - In this activity, we aim that the child learns the letter's ductus by watching the robot moving on a map with the grapheme of that letter. The robot performs the dynamic that should be done while writing with its autonomous motion as the first representation of the letter's ductus. In addition, the letter's phoneme is generated at the beginning and the end of the writing process, to strengthen the link with the corresponding grapheme. The robot's rotary LEDs turn red and blinking, in order, one by one as a progress indicator while the path is followed, and turn solid green when the end point is reached.
- Sub-Activity 2: Link between grapheme and ductus - *Feel the Robot* - While the child watches the robot only in the first activity, we add another representation of the letter's ductus in this second activity by asking the child to put their hand on the robot while it is drawing the letter. The child does not actively move the robot, but only follows its autonomous motion in a passive way. Figure 1A shows an example screenshot of Feel the Robot activity where the child follows her robot with her index finger, while it is performing the ductus of letter “e.”
- Sub-Activity 3: Memorizing the ductus of the letter - *Drive the Robot* - In this activity, the child actively drives the robot in order to produce the ductus of the letter. The grapheme of the letter is drawn on a map as seen in Figure 3A, the design of which includes a car racing theme with the start and end points that the writing should follow. Each child moves with their own speed since the robot is in passively drivable mode. The robot provides assistive haptic feedback by moving the child's hand toward the expected path if the child moves away from it. In order to discriminate the active and passive roles of the children in sub-activity 2 and 3, we assigned different colors to the LED's while robot is on the path. The robot's LEDs are blue while the correct path is followed, turn red if it is out of the letter path and turn green when the end point is reached. These feedback elements condition the child to recognize errors, and serve as extrinsic motivation for drawing correctly. Figure 1B shows an example screenshot of Drive the Robot activity where the child on the left reached the end of the letter “m” (the robot's LEDs turn green) and the child on the right drives her robot on the correct path (the robot's LEDs are blue).
- Team Activity: Recalling grapheme by watching ductus - *Guess the Letter* - In this team activity, children form groups where one child takes turns at drawing a letter with a robot. Each time, the other children have to guess which letter is being drawn. In the group, the two guesser children sit together, with the writer separated from the other two by a physical barrier in order to ensure that they cannot see each other. The writer has one robot, and the two guessers have one robot (or one robot each depending on the size of the workspace) that reproduces whatever movement the first robot performs. In the beginning of the activity, the writer is shown (privately) the map of the letter that indicates only the grapheme, which they then have to draw with their robot. The other children watch their robot reproduce the letter drawn on their empty map. Then, the guessers have to choose the correct letter by recalling the letters they learned or selecting among given graphemes. An illustration of this activity can be seen in Figure 4 where the two children on the left of the barrier are the guessers and the child on the right side is the writer.

#### 3.1.3. Performance Evaluation Design

In order to explore the added value of our system to handwriting learning, the visual perception and the visuomotor coordination aspects should be assessed in detail. Therefore, we focused on assessing the quality of the letter representation in the child's mind in terms of ductus and grapheme. Three sub-skills mentioned above are evaluated in a software application developed in Python that runs on a graphic tablet (Wacom Cintiq Pro). The use of the graphic tablet allowed us to save various data concerning the child's handwriting: The *x* and *y* coordinates of the pen were recorded as well as the pressure and the pen tilt for every time frame at a sampling rate of 200 Hz.

**Figure 5:**
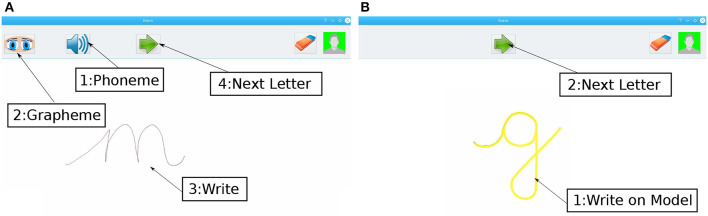
Pre/post-test software: **(A)** Test without letter grapheme to assess the link between the phoneme of the letter and its associated grapheme and ductus. By pressing button #1, the child hears the phoneme of the letter. With button #2, the child has access to the grapheme of the letter during 1 s. #3 is the grapheme drawn by the child. Once finished, button #4 is used to save the data and move to the next letter. **(B)** Test with letter grapheme to assess the link between the grapheme of the letter and its associated ductus. #1 is the letter drawn by the child on the grapheme. Once finished, button #2 is used to save the data and move to the next letter.

- *From Phoneme to Grapheme & Ductus*: In this test, we aimed to assess if the child remembers both the grapheme and the ductus of the letters: The child hears the phoneme of a letter (upon pressing button 1 in Figure 5A) and is asked to draw the grapheme on the tablet. As the link between the grapheme and the phoneme of the letter might not yet be fully operational, we offer the child the possibility to see the grapheme of the letter (only the grapheme and not the ductus) during 1 s, upon pressing button 2. As the child might want to have access to the grapheme even though they have the representation of the letter in their mind (just to make sure they are writing correctly or to ameliorate the letter), we ensured throughout the test that they can press the button only if they have not memorized the grapheme of the letter at all. Since the model of the letter grapheme is not given as default in this test, we referred this pre/post test as *test without grapheme* in this paper.
- *From Grapheme to Ductus*: This test is aimed to evaluate the grapheme-ductus link: The letter's grapheme is displayed on the tablet's screen (see Figure 5B), and the child is expected to draw the letter directly on top of the grapheme. The specific path between the start and end points of the letter is assessed during the test.
- *From Phoneme to Grapheme*: The goal of this test is to evaluate the visual perception which helps the child to find the right grapheme after hearing the phoneme of a letter among other letters. Concretely, the child has to press a button to hear the phoneme of a letter and find the associated grapheme among a choice of given letters.

### 3.2. Initial Testing in Public School: Iteration 2

With the activity and evaluation design done in Iteration 1, initial experiments were conducted with 17 five-year-old children in a public school. The students were split in two learning groups in order to explore the potential benefit of teaching sessions involving the robots compared to teaching session run with more traditional methods. Furthermore, research was done to inspect how these two teaching methods (with the robots and with traditional methods) can be combined together.

Results show a clear potential of our robot-assisted learning activity, with a visible improvement in certain skills of handwriting, most notably in creating the ductus of the letters, discriminating a letter among others and in the average handwriting speed. Moreover, we show that the benefit of our learning activities to the handwriting process increases when it is used after traditional learning sessions. These results were previously published in Asselborn, T.* et al. (2018) in detail; in this paper, we only focus on the insights and observations contributing to future design. Notably, we received the following feedback:

- *Difficulty of Feel the Robot*: The children were frequently having problems in doing this activity due to excessive downward force they applied to the robot which blocked its motion. This required the experimenters to intervene and show the child the proper way to do the activity. Even though initially we decided to abandon this sub-activity in the future designs, discussions with the therapists revealed that the feedback loop provided by the robot not moving while the child is applying too much pressure could be useful for conditioning some children in reducing this pressure. More detail is provided in the corresponding iteration description below.
- *Pre/post-test duration*: Even tough inspecting the learning performance for each learning goal is crucial, collecting data through several pre/post-tests, which must be done for each child participating to the sessions, were observed to be very time consuming. Due to this, we decided to adopt fewer, more focused learning goals in order to be able to design shorter evaluations per session in the future.
- *Confusing visual feedback color*: During the activities, it was observed that one particular child became mad at her robot since it was giving red visual feedback during the View the Robot sub-activity. She said that her robot is misbehaving and not working properly, rightly on her part, since the Draw the Robot sub-activity uses red color to create the negative reinforcement feedback. It was a usability flaw which was fixed for the subsequent iterations.

### 3.3. Adapting the Activity to Occupational Therapy: Iteration 3

#### 3.3.1. Overview

The re-design process comprised of a number of successive iterations with the participation and feedback of several therapists from 2 different therapy centers during 3 summer school activities including multiple groups of children. In this section, the adaptation of the activity to the first therapy center is described, which started with preliminary meetings with therapists in order to do the adaptations specific to the therapy center's teaching methodologies and learning objectives. Taking into account the specific stage the child participants and the therapists were at during this time, it was decided to work on round cursive letters “a,” “d,” and “g.” The previously designed racing theme was kept, as can be seen Figure 3A.

#### 3.3.2. Change in Context, Frequency and Pre/Post-tests Due to Time Limitation

The principal change was on the total time of the activity and limitation of the time spent on pre/post-test evaluation: We decided on less repetition on Watch the Robot, Feel the Robot and Drive the Robot sub-activities and on using only one of the pre/post-tests that focuses on ductus learning evaluation. We chose the From Grapheme to Ductus test since it showed the most clear contribution of our system during Iteration 2.

#### 3.3.3. Removing the Grapheme From the Guess the Letter Activity to Focus on the Goal of Recalling Grapheme & Ductus

Another crucial change done was with the Guess the Letter game activity where the grapheme was removed on the writer side to force the child to remember the letter grapheme, which was hypothesized to be more effective to learn the letter compared to providing the grapheme. Variation between two maps can be compared by checking with grapheme version in Figure 4 and without grapheme version in Figure 1C. This adaptation does not change the learning objective for the guessers but changes the learning objectives for the writer by contributing to the final goal of our learning objective: To encourage the child to remember both grapheme and ductus. If the writer cannot write the letter properly, by definition the guessers cannot guess correctly. This becomes a feedback mechanism for the writer to rewrite the letter by paying better attention to the writing process. Since the grapheme is not there anymore, it further allows us to track the progress of the child through the writing trajectory data which does not necessarily follow the correct path. Some example trajectory results of this feedback mechanism are given in section 4.2.

### 3.4. Testing in the First Therapy Center: Iteration 4

The activity is tested within the first day of the summer school with 5 boys for 1 h. The information related to this group can be seen in Table 1 - Group 1. We encountered a number of problems in the pre/post-test application, gathered observations that highlight the added value of the activity, and feedback from the therapists, which are summarized as follows:

- *Problem of sequential testing design*: The activity is started with testing with the grapheme. After second child's tests, the rest of the group were bored of waiting for 10 min and the pre-test was not completed. The sequential design (pre-test one child at a time) did not work due to the limited attention span of the target child group. For the following summer school sessions we decided to do the testing while the other children doing another group activity and not waiting for each other.
- *Added value of the Feel the Robot activity for sensing self-applied force*: Child F has the problem of discriminating the relationship between his touch sensation and visual perception. Therapists indicated that Feel the Robot activity is very useful for children having such problems to train on exerting the right amount of force by improving the connecting between sight and touch sensations. As it is observed with Child F, while the robot was blocked by putting too much force on it, in order to observe the motion of the robot, the child was encouraged to balance and reduce this force. In doing so, he was training in controlling it.
- *Motivation and engagement*: The overall group motivation was observed to be high and the attentive time spent on our activity was observed to be longer compared to other writing activities. In particular, the total time child F was attentive was considerably high according to therapists, since he does not like to write and he did not previously focus on a writing task for such a long period of time. He was observed to be highly willing to write with Cellulo and he readily completed all of the tasks.

### 3.5. Adapting the Activity to the Second Therapy Center: Iteration 5

#### 3.5.1. Overview

Apart from the necessities of integration to the occupational therapy environment, it was observed in the previous iteration that there may be a need for further adaptation to each therapy center to be compatible with their learning methodologies. In this iteration, besides doing this, we also integrated the previously suggested changes by therapists to our activity design and flow. These are discussed below.

#### 3.5.2. Re-designing Visual Cues to Be In-line With the Present Teaching Methodology

The main change in this iteration was to adapt the cursive letter shapes and visual cues on the map designs as the way of teaching cursive letters differed from the previous therapy center: The letters were adapted to also include the connecting strokes from the previous (imaginary) letter as the initial stroke, which was not previously present in our design.

Furthermore, the methodology designed by the ABC Boum+ company^4^ was adopted as it was being used in the therapy center. This method combines visual, conceptual and sound cues with the initial strokes of the cursive letters in order to reinforce the learning, where the letters are divided into different conceptual groups according to their initial strokes. We designed new maps with these new cues instead of the car racing theme, except the trophy icon at the end which was kept. We also designed new maps consisting of only the cues to teach the initial stroke of the corresponding letter group. Three example map designs from “wave letters” and “skateboarding letters” groups and their corresponding initial cues can be seen in Figures 3B,C, respectively.

#### 3.5.3. Knowledge Transfer From “Large Letters With the Robot” to “Small Letters With the Pencil”

In order to reinforce the ductus and grapheme representation learning, therapists suggested to add writing activities with a pencil and post-it after each letter practiced with the robot. The second reason for this addition was to switch between a gross motor activity to a fine motor activity to help mapping the learned shape to actual handwriting practice. This allowed us to confirm whether the writing practice with the robot, with the pencil and with the ThinkPad pen are similar or not. See section 4.4 for writing performance comparison and discussion.

### 3.6. Testing in Second Therapy Center: Iteration 6

**Figure 6:**
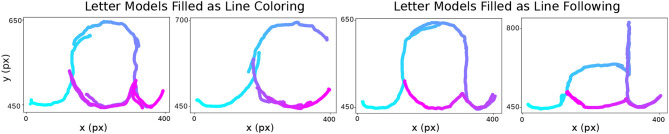
Mis-perception of the letter models, the time dimension in the data is indicated by the color of the stroke going from light blue to light pink: Some children filled the grapheme of the letters as if it is a line coloring/painting activity (on the left, note the strokes going back and forth), or a line following activity (on the right, note the strokes following the grapheme's lines continuously but not in the correct ductus) in the pre/post-test with the grapheme.

The activity was tested during the each day of the first summer school (3 days) with 3 girls. The information related to this group can be seen in Table 1 - Group 2. Our findings are as follows:

- *Effect of summer school context on engagement*: For the first day of the activity, 4 letters were selected, which made the activity length roughly 50 min in total. This duration was quite long in comparison with the duration of the other activities within the summer school. Since it was a summer school including both gross and fine motor skills, there were several active game-play sessions including games in the playground, jumping, climbing etc. Within this context, the duration of the activity with the robots played a crucial role to keep the attention and engagement of the children stable. For instance, child J did not want to continue the activity because the other activities were more fun in her view. For this reason, from the second day on, we reduced the number of letters to 3 in the activity, which reduced its length to roughly 40 min.
- *Group therapy including children with different abilities*: Child I (age 5.5) was younger than the rest of the group (mean age 7) and she had not received any lesson on cursive writing before our activity. Even though it was her first time writing cursive letters, she was observed to perform well albeit with the help of graphemes provided to her on a post-it, which was not given to other children. Child C has problems with fine motor skills, attention, and organization. Therapists indicated that sometimes while writing she is not totally concentrated or focused. She engaged a lot during the robot mediated activities and liked the game. Each day she wanted to continue to do more exercise with the robots.
- *Pre/post-test mis-perception*: The most discriminative test previously used (writing on top of a grapheme, i.e., From Grapheme to Ductus) that showed best the progress of the healthy children in the school context did not work with some children in this therapy center. These children did not understand the relationship between these letters and the activity, and proceeded to fill the letters as if it was a line following task (see Figure 6 for sample data): The graphemes on the screen were not perceived as a letter writing grapheme but as a line to be followed and/or an area to be painted. Therapists suggested that if there is no stable grapheme in the test, it might be easier for the children to avoid this confusion. Therefore, from the second day on, we switched to the pre/post-test without a stable grapheme where grapheme was made to appear on the screen for only 1 s after pressing the grapheme cue button (i.e., From Phoneme to Grapheme & Ductus).

### 3.7. Second Testing in Second Therapy Center: Iteration 7

The activity was further tested during the first 2 days of another summer school approximately 1 week later, with 4 boys. The information related to this group can be seen in Table 1 - Group 3. We found that:

- *Difficulty of changing the letter maps during the session*: Even though the therapists found the activity useful for children, it was observed that it is difficult for one single teacher/therapist to control the whole activity flow including several letter maps in a session with 4 children. For future use in such group sessions, they proposed using large, thick sheets of paper or paper sheets attached to thin wooden blocks for further ease of changing maps.
- *Need for practice in recalling the grapheme*: Most appreciated feature of the activity by the therapists was having separate sub-activity alternatives, with and without grapheme. It was suggested that a version of the Drive the Robot without the grapheme (i.e., empty map, similar to how it is done in the pre/post-test and the Guess the Letter game) should be added alongside the one with the grapheme, in order to provide an exercise in recalling the grapheme of letters.
- *Loss of motivation due to passive tasks*: In the second day, one child was observed to lose engagement in the Guess the Letter game while he is in the role of guesser which resulted in inattentive and random guessing, as he indicated that he would like to play the writer role instead. This implies that, to be more robust against such cases, more variants should be done to ensure that every sub-activity and every role could be tweaked to include active participation.
- *Need for repeated sessions*: Since children learn script letters before the cursive letters at school, the change could be difficult for them, as expected. Therapists reported that indeed more repetition of the sessions is needed before the ductus knowledge could be fully integrated.

After this session, we also had the chance to get the children's feedback on which part of the game they like the most and the least:

- Child K: He enjoyed every part of the activity, particularly the Guess the Letter game.
- Child O: He enjoyed Guess the Letter game the most and Drive the Robot the least.
- Child S: He enjoyed drawing the letter during Drive the Robot the most, while he enjoyed the rest of the activity in general. He was very attentive during both days and even named his robot.
- Child M: He enjoyed the writer role in the Guess the Letter game the most, but enjoyed the guesser role the least.

## 4. Results

**Figure 7:**
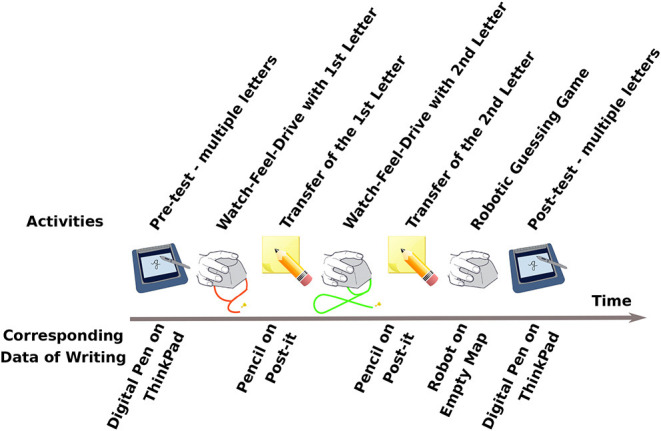
An example activity flow of a robot-assisted writing session with two letters and corresponding handwriting data collected with a different medium in each step.

Our iterative design approach allowed us to successfully integrate our robot assisted writing activity into the occupational therapy center by adapting the activity as well as the evaluation methods for different therapy centers and groups. During this process, a number of different letters are practiced with our robotic platform during several sessions. In this section, we first investigate the effectiveness of our activity in teaching the child participants to write letters. Second, we focus on the effect of changes made during the iterative processes on the writing performance. An example activity flow of a refined robot-assisted writing session and corresponding handwriting data collected with different mediums in each steps can be seen in Figure 7.

### 4.1. Overall Learning

**Figure 8:**
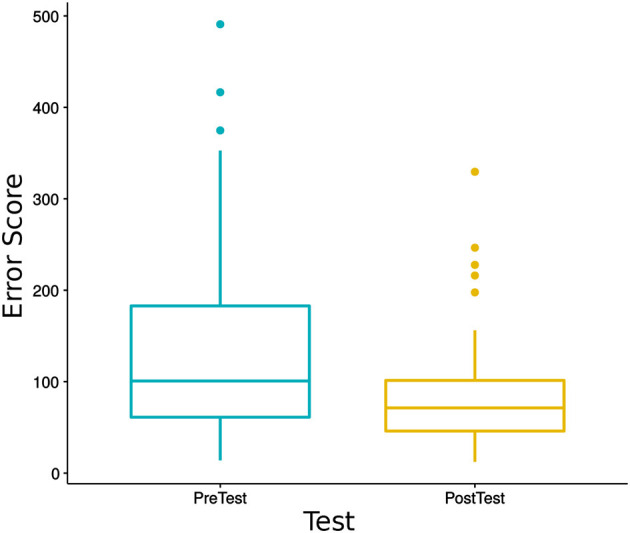
Comparison of DTW error scores of all children for pre-test and post-test. We found that post-test error scores are significantly lower than pre-test error scores (*V* = 1449, *p* < 0.001).

During each iteration, before and after the learning session, a pre/post-test is done to measure the progress in letters learnt during the sessions. To analyze if there was overall learning in writing letters for all sessions for all children, we did a Wilcoxon Signed-Ranks Test, which indicated that post-test error scores were significantly lower than pre-test error scores [*V* = 1, 449, *p* < 0.001, *r* = 0.42 (moderate effect size)], see Figure 8.

**Figure 9:**
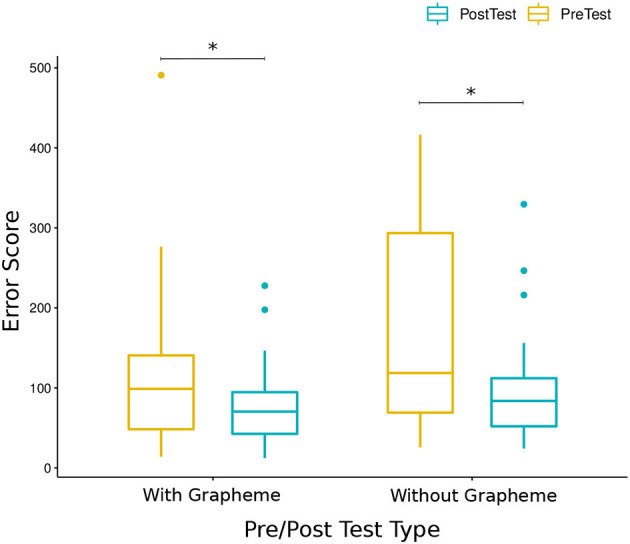
Comparison of DTW error scores of all children for pre-test and post-test using the test with grapheme and the test without grapheme. In both with and without grapheme tests, post-test error scores are significantly lower than pre-test error scores (*V* = 160, *p* < 0.05, *r* = 0.45 and *V* = 681, *p* < 0.01, *r* = 0.65).

Since two types of pre/post-test evaluation are used to measure overall learning, we also checked for per-test learning by doing two separate Wilcoxon Signed-Ranks Tests. In the data collected with *the test with grapheme* during Iteration 4 and the first day of Iteration 6, we found a significant decrease in error scores of post-test compared to error scores of pre-test [*V* = 160, *p* < 0.05, *r* = 0.45 (moderate effect size)]. Similarly, in the data collected with *the test without grapheme* during day 2 of Iteration 6 and Iteration 7, we found significant decrease in error scores of post-test compared to the pre-test [*V* = 681, *p* < 0.01, *r* = 0.65 (large effect size)], see Figure 9.

In order to test if there is any significant difference between the average performance of the children in the 3 experimental groups, a Kruskal-Wallis Test was done which revealed no significant difference between groups (*H* = 0.17, *df* = 2, *p* = 0.92).

**Figure 10:**
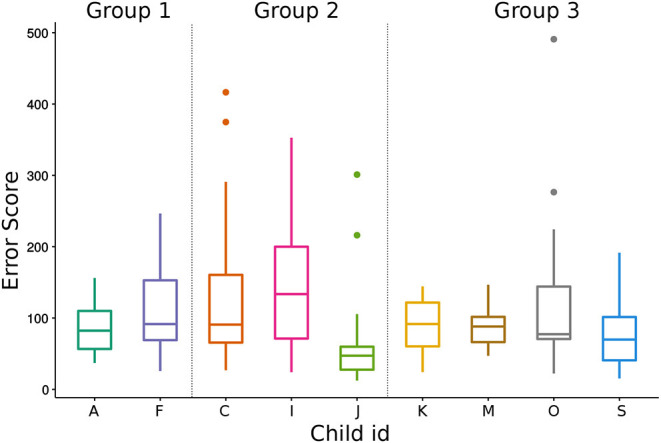
Total pre/post-test error score of each child (Excluding three the children in Group 1 who could not attend pre/post tests due to the time limitation).

Similarly, we checked child-level difference in overall data including pre/post-test error scores with Kruskal-Wallis Tests and found significant difference (*H* = 18.91, *df* = 8, *p* < 0.05). In order to identify which pairs of children is different from each other, we did multiple pairwise comparisons between children with a Pairwise Wilcoxon Test and found that error scores of child J is significantly lower than child I (*p* < 0.05), please see Figure 10 for the comparison.

In order to test if there is a significant difference between children in pre/post-test score difference (improvement in writing), we did a Kruskal-Wallis Test and found no significant difference between the improvements of children (*H* = 8.66, *df* = 8, *p* = 0.37).

### 4.2. Effect of Removing the Grapheme From Guess the Letter Game on Writing Performance

**Figure 11:**
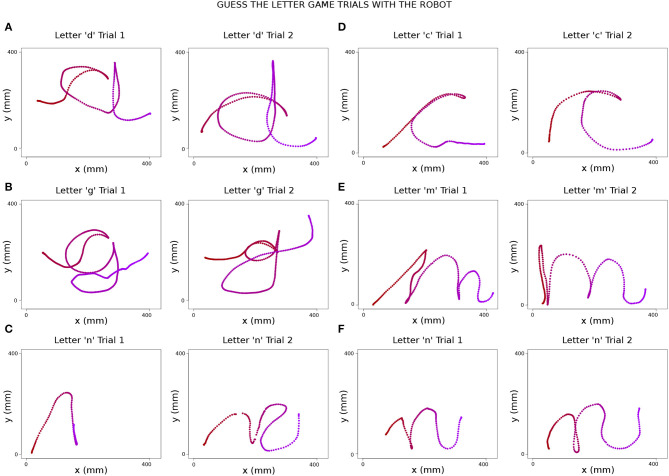
Sample improvements in Guess the Letter game trials. The time dimension in the data is indicated by the color of the stroke going from dark red to purple. **(A)** Trials of writing “d.” On the second trial, the child made the upper tail of “d” longer to differentiate it from “a,” which was the previous answer from the guessers. **(B)** Trials of writing “g.” On the second trial, the child made the curved tail of “g” rounder to be more clear for the other children who were guessing. **(C)** Trials of writing “n.” In the second trial, the child wrote a better version of “n” by paying attention to the cursive start. **(D)** Trials of writing “c.” On the second trial, the child made the “c” more curvy. **(E)** Trials of writing “m.” In the second trial, the child wrote a better version of “m” by paying attention to the proportional size of its different parts. **(F)** Trials of writing “n.” The child wrote a better version of “n” in the second trial by paying attention to the direction of the lines and cursiveness.

In the initial version of the Guess the Letter game, the writer did not have to reflect on the writing performance as he/she had the grapheme available directly on the activity map. Upon removing the grapheme, the writer was obliged to listen to the feedback given by his/her guesser friends in case they did not understand which letter is drawn due to poor writing. This forces the writer to pay more attention to the discriminative features of letters. To this feedback mechanism, the therapist sometimes contributes additional cues such as, “Write it bigger,” “You should make the tail longer,” etc. Figure 11 displays the sample letters written during the Guess the Letter game. Letters indicated as “Trial 1” are the first writing trials of the children which are not understood or not guessed correctly by their peers. Letters indicated as “Trial 2” are the second writing trials of the children just after getting feedback from peers and therapist on the first trials.

### 4.3. Effect of Removing the Grapheme in Pre/Post-tests on Number of Strokes

**Figure 12:**
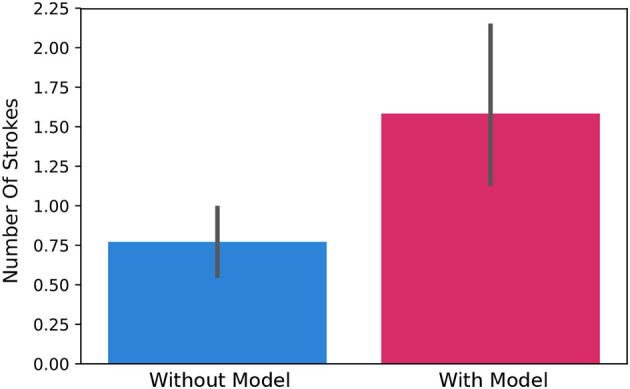
Total number of strokes to write a letter with the test not including a grapheme (on the left) and with the test including the letter grapheme (on the right). We found that the number of strokes is significantly lower in test without the grapheme than in the test with the grapheme (*U* = 828.5, *p* < 0.01).

Pre/post-test type is changed during Iteration 6 due to the mis-perception of the test with the grapheme. In order to see the effect of this mis-perception on handwriting quality, connectedness of each letter is calculated by counting the number of strokes used to write each letter. We did a Mann-Whitney *U*-Test to compare the number of strokes to write a letter in the test with grapheme and in the test without grapheme. We found that number of strokes is significantly higher in test with grapheme (*U* = 828.5, *p* < 0.01), meaning more connected letters were drawn when the grapheme is not provided, see Figure 12.

We also looked for the change in number of strokes before and after the writing activity. A Mann-Whitney U Test is used to compare the pre-test and post-test in the number of strokes (using data from both the test with grapheme and the test without grapheme). We found that the change is not affected by the test type (pre-test v.s. post-test), and there is no significant difference in the change of number of strokes (*U* = 290.5, *p* = 0.31).

### 4.4. Knowledge Transfer

**Figure 13:**
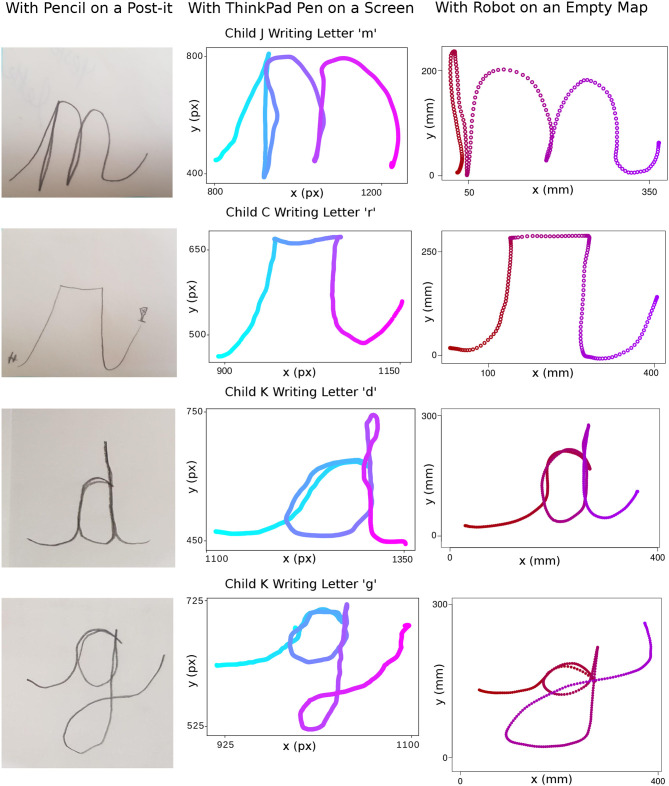
Samples of letters “m,” “r,” “d,” and “g” written with a pencil on a post-it, with ThinkPad pen on a screen and with the Cellulo robot on an empty paper map. The time dimension in the data coming from ThinkPad pen writing is indicated by the color of the stroke going from light blue to light pink. The time dimension in the data coming from the robot writing is indicated by the color of the stroke going from dark red to purple.

**Figure 14:**
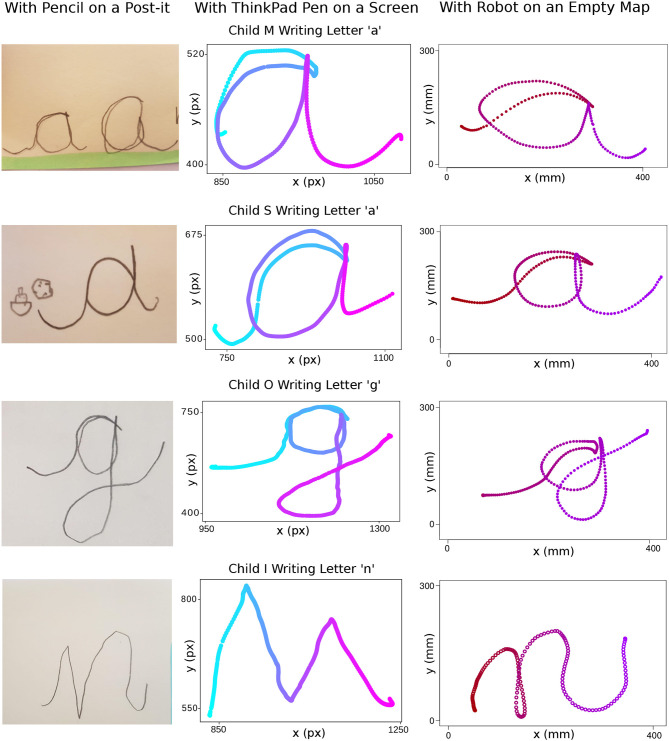
Samples of letters “a,” “g,” and “n” written with a pencil on a post-it, with ThinkPad pen on a screen and with the Cellulo robot on an empty paper map. The time dimension in the data coming from ThinkPad pen writing is indicated by the color of the stroke going from light blue to light pink. The time dimension in the data coming from the robot writing is indicated by the color of the stroke going from dark red to purple.

In Iteration 5, in order to switch between gross and fine motor activities after each letter practice with the robot, the therapists suggested to let the child write the letter in focus with a pencil on a post-it. This also allowed us to monitor the differences and similarities between the writing practice and performance with our robot on an empty map, and with a pencil on a post-it. In this comparison, we also included the writing on the tablet screen with its pen, used in the pre/post-tests, in order to compare and contrast our evaluation practice against the actual writing task. Sample letter performances in this comparison can be seen in Figures 13, 14.

In all three variations of the given samples, there is general consistency in the grapheme and ductus. Nevertheless, it is clearly visible that there is increased jerkiness of motion in most of the letters written with the tablet pen compared to the ones written on paper and written with the robot. There are further slight differences between the methods, such as alignment problems with “a” and “g” in the case of the robot. Finally, the letters written by Child I were observed to be inconsistent in general, which may be due to several reasons including the child's age, her current stage of learning and the nature of the letters. See the corresponding discussion points below on each of these observations.

## 5. Discussion

### 5.1. Overall Learning

The presented activity is designed to support children in learning to write cursive letters within occupational therapy sessions. Reported experimental results suggest that children having writing problems are able to improve in letter writing after the use of the system for one session. This was evident by an overall significant decrease in error scores of post-test compared to the error scores of pre-test.

Furthermore, while investigating individual performance differences per child, we found that only the performance of child J and child I were significantly different than each other. As can be seen in Table 1, child I was the youngest participant, having her first experience with cursive letters, while child J has the overall best performance and high intelligent assessment.

The score data probing the learning gain differences per child show that even though the levels of the children are different, the learning gains in handwriting are similar, thus suggesting that the activity is inherently adaptive to the learner's abilities and expertise.

### 5.2. Effect of Removing the Grapheme From Guess the Letter Game on Writing Performance

As results in section 4.2 reflect, providing another version of the game by removing the letter grapheme from the writer side allowed children to learn from their errors when their peers could not guess the correct letter. Here, adaptive content of the game allowed us to change the learning goal of the game for the writer, i.e., remembering the ductus in the version with the grapheme v.s. remembering both the grapheme and the ductus in the version without the grapheme. In the version without the grapheme, we observed that the writer child was encouraged to focus on the proportionality of the letter's parts, as well as its discriminative parts from other letters. For instance, in Figure 11, the first trial of letter “d” was perceived as an “a” by the guessers and the writer prolonged the upper tail of the letter “d” to make it distinguishable from an “a.” The sequence of “n” letters written in the second trials shows the importance of paying attention to the starting gesture and direction of the strokes belonging to the letter.

Even though the learning objective for the writer is changed for the occupational therapy, if desired, within the session, the version with the grapheme can be rapidly switched to, in the case where the learning objective is the ductus only, e.g., in case of a very preliminary learning stage.

The new Guess the Letter game version can also improve children's understanding by their peers in successive trials. Peer collaborative interactions are crucial for a child's learning: Vygotsky (1980) stated that learning awakens in children a variety of internal developmental processes that can operate only when they interact with more competent people in their environment and in cooperation with their peers. The effect of removing the grapheme placed onus on both participants in the Guess the Letter game, brought cooperation to the forefront and was supported by the therapist cues - all highly benefiting the writer in enhancing their learning.

### 5.3. Added Value of Adaptive Content

The behavioral observations and feedback of therapists through the iterations emphasized the importance of using adaptive interfaces. The unique localization mechanism of the Cellulo platform allowed us to switch from with grapheme to without grapheme versions of the sub-activities, easily adapting to different learning objectives for different letter representations.

The ability to change the number of letters to be learned during sessions and between sessions enabled adapting the activity flow and the total time of the therapy session. We were able to thus tune the duration of Guess the Letter and the total number of times Watch, Feel and Drive the Robot sub-activities by taking the motivation level of the children into consideration. This temporal adaptivity is learned to be crucial in a therapy setting: We experienced a number of failures due to the previously fixed natures of the activity in various iterations, which had been successfully applied previously in a public school environment with typically developing children. In this case, the limited attention and concentration spans of some children in need of such therapy in handwriting absolutely requires this kind of adaptivity; otherwise the activity risks failing at some point. From another perspective, different therapy centers vary in their availability in time and this availability is typically very limited. These two facts further emphasize the importance of temporal adaptability in enabling applicability in a large number of therapy centers, as opposed to being targeted to the scheduling and practices of a single collaborating center.

Adding traditional paper-based activities between each robot-assisted letter activity allowed us to encourage mapping between large and small letters, and between the writing tool used i.e., robot and pen, while allowing us to switch between training gross motor skills and fine motor skills. This change also allowed us to compare and contrast the performances while using different writing media. This is another form of adaptivity of our system design providing another kind of added value, namely adaptability and especially flexibility for integration with traditional practices, which we have previously shown to be potentially useful in improving the gain from the robot-assisted activity (Asselborn, T.* et al., 2018).

### 5.4. Added Value of Robotic Platform Capabilities

The Cellulo platform allows us to implement parallel robot behaviors where the tasks for different children can be orchestrated simultaneously within the same activity. By scaling up/down the number of robots, the activity can serve to groups of different number of children where one robot can be assigned to one child and be programmed to do the same task as all other robots do. This feature allowed us to design three such sub-activities: Watch, Feel and Drive the Robot.

Tests conducted in therapy environments highlighted an advantage of another feature where a robot becomes active when the corresponding child puts his/her robot on his/her paper map, since the localization is dependent on the paper. This allows parallel activity flow that is compliant to each child's attention and intention to start.

The Cellulo platform also provides synchronized robot behaviors where the behaviors of each robot can depend on each of the other robots, i.e., provides swarm behaviors. Using this attribute, we designed the Guess the Letter game where the writer robot guides the rest of the robots. In different therapy centers and sessions, we had varying room settings according to the availability of dissimilar rooms and tables with varying number of children attending the session. Synchronous and parallel capabilities of the robots enable parallel and/or shareable activity workspaces where we can group or separate children, distributing them to different tables with different workspaces as needed. Therefore, the system can be adapted to: (1) The unique room settings of different therapy centers involving the size of the tables, the number of tables and the type of the divider preventing the guessers from seeing the writer during the Guess the Letter game; (2) Number of children attending the session.

### 5.5. Effect of Pre/Post-test

After using a pre/post-test targeting the measuring of three different learning objectives in Iteration 2, we found that integrating all of these objectives into the therapy environment may not be feasible due to time limitations. Therefore, one test among them that most strongly emphasizes the added value of our activity was selected for use in therapy sessions. However, during the sessions of Iteration 6, the test was mis-perceived as a line following or coloring activity by some children.

This is evidence that perception of such activities by children with attention or visuomotor coordination problems might differ from typically developing children. Even within each group, there may be differences on perception and mapping capabilities. As results in section 4.3 indicate, the device or medium used for pre/post-testing may affect the resulting performance, simply because of this mis-conception of the provided test design. Therefore, for each special group, the system should be able to provide alternative pre/post-test design choices and the designers should question whether they can use the pre/post-tests which are typically designed for regular schools in a special education setting.

### 5.6. Effect of Writing Tool and Knowledge Transfer

Accommodation of the hand and the grasping and moving styles were different in each medium. Typically for the screen, there were unintended touch events caused by resting one's palm or grazing the fingers over the surface. Presumably because of this phenomenon, some children were observed to adopt an uncomfortable writing position to avoid the unintentional touch event. Another reason for this observation might be the dissimilarity between the friction provided by the tablet surface and its pen, and the typical friction provided by pen and paper surface. A previous study (Annett, 2014) reported that many participants felt that “there was not enough friction between the pen and screen to feel natural” and their hand jerked across the screen as they moved it. This mismatch was also reflected in the number of participants who floated their palms above the surface of the screen which might be due to the different feeling of new pen and screen friction type different than the friction between pencil and paper.

Even though there is jerkiness of motion with the digital pen and screen, the letter shapes in our case are observed to be similar to the ones written on paper, even when we take into account that children are used to writing with pen on paper as typical handwriting practice, and that a digital pen is a new medium for them. Furthermore, the data and the information that a digital platform provides is very valuable from the perspective of teachers and therapists: For instance, this data can be easily made to reflect if the child knows the grapheme and ductus by providing direction information with color coding. For this reason, it must be considered by the designers of handwriting learning activities whether this jerkiness of motion is an important factor or not, and whether it disallows the use of tablets, depending on the needs of the specific application.

In the robot medium, children are using the whole hand to grasp the robot which makes the practice more comparable to gross motor action supported by arm motions, where it is typically easier to control the writing action. This may be a strong reason why we do not observe jerky motion in robot writing. From another perspective, teachers indicated that it is very promising how children can reflect the knowledge of writing onto a robot, which is to a certain extent different than other typical school activities including writing with finger, with pen or with pencil: The robot appears to support the skill transfer from pen and pencil, where all the letters look like written letters when viewed in the same size. This indicates the potential of the robots as an interesting alternative approach providing more feedback than a traditional sandbox or home remedies.

Even though there is a lack of visual feedback of the written letter with the robot (the robot does not leave any “ink” on the paper), the letters written with the robot were observed to be of similar quality to the ones written with the pen on paper. However, the alignment of the strokes which pass over or under previously drawn strokes were more difficult to adjust on an empty map since the previously drawn part of the letter cannot be seen visually. This results in typically more disproportionate parts in letters involving such strokes, such as “g”: This is exemplified in Figures 13, 14, which also show a similar problem in the letter “a” whose initial connecting stroke is typically more disproportionately positioned compared to pen and paper where the strokes can be made to pass exactly on top of each other more easily.

Comparing the writing of the letter with a pencil on a post-it, then with the robot on an empty map, and finally with a tablet pen on a screen, gives a useful picture to the child's strengths and points where he/she is having difficulties: Fine motor skills, gross motor skills, child's preference of the tools etc. By learning more about where an exact difficulty or strength may be for given child, a therapist or teacher can add more tools and options to support him/her with overcoming his/her writing difficulties. Furthermore, it gives a variety and interest to the practice that pencil and paper alone cannot provide. It provides an opportunity for the child to work with their favorite writing tool and transfer the grapheme and ductus skills to a less favored tool.

From an individual child level, only the performance of the 5.5-year-old Child I was observed to be inconsistent across the writing tools. This may be due to the lack of orthographic coding of the letter “n” in this child which facilitates forgetting the grapheme of the letter, which could have occurred at different points in time within the activity. It could also be letter dependent since the cursive letter “n” has repetitive bumps making it harder to consistently reproduce. It may be found by future studies that the expectation of mastery in the cursive grapheme and ductus at this age is not be feasible at all.

### 5.7. Activity Presentation and the Overall Theme

A child who does not enjoy writing was totally engaged for 40 min of the session that was presented within a writing theme (Child F), whereas another child totally lost attention after 30 min of the session that was presented in a more physically active theme (Child J). Here, we observed that the general theme of the therapy session may drastically affect the perception of the proposed activity. For instance, if the robotic writing activity is part of a general writing session, it may boost the child's motivation and engagement. However, presenting it among other activities involving games with more physical activity where children can run, jump, climp etc. can make it more difficult and less motivating for the child to sit down and concentrate for 40 min. Therefore, the general theme within which the writing activity will be proposed should be considered carefully when designing the activity flow, and its duration and composition should be adapted accordingly.

### 5.8. Novelty Effect

Even though the engagement was observed to be very high for our studies which took 2–3 days, it is not realistic to expect efficiency and engagement in the long term because of the well-known novelty effect typically associated with technologies such as ours. For overcoming this challenge, we hypothesize that the activity could be extended with new drawing concepts and free-drawing sessions. These sessions may include drawing any geometrical shapes, numbers, animals or objects with their model visible in the Feel and Drive the Robot sub-activities or the Guess the Letter game. Guess the Letter can also be modified by various themes, such as:

- Writing the first letter of a friend's/object's/animal's name and guessing who/what it is.
- Free-drawing where the writer child can draw anything they imagine without necessarily using a model: A toy, a house, an umbrella etc.
- Writing the initial cue of the letter (such as wave) while the guessers guess the group of possible letters (such as “a,” “c,” “d,” and “g”) as a more advanced sub-activity.

### 5.9. Limitations and Future Work

Even though writing on an empty map pushes the child to remember and practice what they learned before, the robotic platform lacks visual feedback since it cannot provide the visual output of what is previously drawn by the child. The only feedback is the peers' perception of the writing and the therapist's cues such as: “Your friends didn't understand what you drew, you should write it bigger” or “Please write it as cursive as we learned today.”

Another practical limitation of the system is the need to secure paper sheets to the tables, typically done with non-permanent adhesive such as masking tape. For a group of children having attention problems, this alone may create a need for a second therapist since they lose attention quickly while waiting for a preparation process even though it lasts only a few minutes. An alternative is to involve children themselves in this process and having them aid in the preparation and application of the tape, which may also be argued to promote fine motor activity.

The results show the overall effect of the system on progress to handwriting quality of 9 children with visuomotor coordination and attention problems (excluding 3 children whose test data were incomplete). Since the main purpose of the study was to adapt the system to the environment rather than adapting the therapy to the proposed robotic activity, we had a heterogeneous group of children which was a natural aspect of a group therapy session in an occupational therapy center. In order to have more generalizable outcomes, the activity should be further tested in different institutions with more children ranging in age and in difficulties they have. The overall effect of the refined robotic activity (through iterative design process) should be compared with a traditional training process in therapy centers within a study similar to the one conducted in the preschool study. Further research is also needed to investigate the long term improvement and retention in ductus and grapheme learning in such children.

Comparing the results of the children in the therapy center with those of the children in preschool would give us precious insights on the value of the activity with the robot. However, the designs of the activity carried out within school and within the iterations at the therapy centers differ on a number of crucial variables including total duration of the writing activity, mean age of children and device that is used for pre-post test, which reduces the validity of a comparison between the data already collected. Therefore this comparison should be considered for a more controlled follow-up study specifically targeting this question.

The results comparing different writing media and investigating knowledge transfer are limited to observational inputs. In order to explore the knowledge transfer more in depth, more experiments should be conducted where the focus is on transfer learning with quantitative methods.

## 6. Conclusion

Robot-assisted activity was integrated to different occupational therapy sessions and was shown to improve the letter writing performances of children with visuomotor integration and attention problems. This emphasizes the Cellulo interface as a potential tool to conduct handwriting training to teach ductus and grapheme of the letters in multi-child special education environments.

The effective integration of the robot-assisted system into the occupational therapy environment demanded variants of different content throughout the iterations. These modifications included the adaptation of the duration of sub-activities, adaptation of the number of letters and repetitions of each letter in these sub-activities, adaptation of the map graphemes and themes, adaptation of map content (with/without grapheme) within the game, adaptation of game duration and adaptation of activity flow, selectively integrating with traditional practices. These adjustments assisted the consistency and overlap of the learning goals determined by the therapists and the learning goals of the activity. They also allowed adequate engagement of the different groups of children while fitting into the typical timeframe of an occupational therapy session.

## Data Availability Statement

The datasets generated for this study are available on request to the corresponding author.

## Ethics Statement

The studies involving human participants were reviewed and approved by The Human Research Ethics Committee (HREC) at EPFL. Written informed consent to participate in this study was provided by the participants' legal guardian/next of kin. Written informed consent was obtained from the individual(s), and minor(s)' legal guardian/next of kin, for the publication of any potentially identifiable images or data included in this article.

## Author Contributions

AG, TA, and EY: conception and design of the activity, data collection in school, and literature search. TA and AG: design and implementation of the evaluation tests. AG: iterative design and data collection in therapy centers and system design and implementation. AG, WJ, and TA: statistical data analysis. AG, AO, WJ, BB, and MS: interpretation of the results. AG, AO, and BB: drafting of the manuscript. AG, AO, PD, and BB: critical revision of the manuscript for important intellectual content.

### Conflict of Interest

The authors declare that the research was conducted in the absence of any commercial or financial relationships that could be construed as a potential conflict of interest.

## Footnotes

^1^https://www.yourtherapysource.com/blog1/2019/04/24/tips-for-successful-pediatric-group-therapy-sessions/ (accessed November, 2019).

^2^Such as the one in https://www.etsy.com/listing/453872176/cursive-alphabet-wood-tracing-board (accessed September, 2019).

^3^Described in https://childhood101.com/sensory-play-ideas-games-to-develop-the-sense-of-touch (accessed September, 2019).

^4^ABC Boum teaching approach of graphomotricity, https://abcboum.net (accessed September, 2019).
